# SLE risk alleles and cell development and activation

**DOI:** 10.1186/ar3942

**Published:** 2012-09-27

**Authors:** SJ Kim, N Manjarrez Orduno, PK Gregersen, B Diamond

**Affiliations:** 1 Feinstein Institute for Medical Research, Manhasset NY 11030, USA

## 

Understanding the functionality of lupus susceptibility alleles may best be done using cells of healthy individuals harboring one or two copies of the susceptibility allele. With this approach, confounding variables introduced by disease and medication are avoided. We have studied the SLE susceptibility allele of Blimp-1 in dendritic cells and Csk in B cells. The Blimp-1 risk allele exhibits low expression and leads to an increased secretion of proinflammatory cytokines. The Csk risk allele leads to enhanced expression and an increased response to B-cell receptor signaling. These effects of the risk alleles on activated or resting cells, respectively, provide insight into disease pathogenesis.

